# How COVID-19 affects patients receiving anticytokine and JAK inhibitors in rheumatology and dermatology

**DOI:** 10.2217/imt-2020-0153

**Published:** 2020-07-09

**Authors:** Ting Yi Jessica Chang, Janet E Pope

**Affiliations:** ^1^Schulich School of Medicine & Dentistry, University of Western Ontario, London, ON N6A 3K7, Canada; ^2^Division of Rheumatology, St Joseph’s Health Care London, London, ON N6A 4V2, Canada

**Keywords:** antimalarials, coronavirus, COVID-19, IL12/13, IL17, IL23, IL6 inhibitor, immune-mediated diseases, immunosuppressive, SARS-CoV-2

Coronavirus disease 2019 (COVID-19) is an infectious respiratory disease caused by SARS coronavirus 2 (SARS-CoV-2) . The disease was first identified in Wuhan, China and the WHO was alerted in December 2019. By May 2020, the WHO reported over 4.6 million confirmed cases in over 200 countries around the globe [1]. Most individuals that test positive for COVID-19 experience mild symptoms with fever, fatigue and dry cough as the most common symptoms. However, due to a large number of infected individuals, there have been over 313,000 deaths worldwide as of 18 May 2020 [1]. As a result of the widespread disease, many countries have placed limits on travel and social gatherings, as well as closed schools and nonessential workplaces. The impact of the pandemic affects everyone, ill and healthy, in all aspects of daily living. This article discusses how the COVID-19 pandemic may affect rheumatology and dermatology patients with immune-mediated diseases who are receiving anticytokine or Jak inhibitor therapies.

## Safety of immunotherapy in patients with rheumatic diseases during COVID-19

Patients with rheumatic diseases are often on immunosuppressive medications, and both the disease and the medications contribute to a higher infectious risk, leading to concerns of increased risk and severe disease with COVID-19 in these patients. The concerns have resulted in changes in patients’ treatment plans. For example, an email questionnaire surveying 7061 patients in FORWARD, the US wide registry for rheumatic disease, received responses from 530 participants, of those, 471 responded to questions about changes in their rheumatology care in the previous 2 weeks [2]. Of the 197 patients (out of 471) who reported some change, 14% had made changes to their own medication list or dose while 11% made such changes based on physician recommendations [2]. The survey identified the perception of increased risk and potential severity of COVID-19 secondary to taking immunosuppressive medications as a reason for both self-imposed and physician-approved discontinuation of disease-modifying antirheumatic drugs (DMARDs) therapies [2].

Interestingly, preliminary data suggest that patients with rheumatic diseases treated with immunosuppressive agents are not at an increased risk for poor outcomes for COVID-19 compared with the general population. For example, of the 458 patients with rheumatic diseases (56% were on corticosteroids, 44% on conventional synthetic DMARDs and 41% on biologic DMARDs) interviewed in Tuscany, Italy, only 13 reported symptoms consistent with COVID-19 [3]. Of those, seven patients were given nasopharyngeal swab and only one was positive, resulting in a prevalence of 0.22% (0.01–1.21%) which is similar to that of the general population of Tuscany (0.20% [0.20–0.21%]) [3]. Likewise, a survey conducted in Milan, Italy, with 123 adult patients with rheumatic diseases (64.2% were on corticosteroids, 59.3% on conventional synthetic DMARDs and 20.3% on biologic DMARDs), had only one patient with a positive COVID-19 nasopharyngeal swab, resulting in an incidence of 0.81% which is comparable with the incidence of 0.62% seen in the Lombardy region during the same time period [4]. Similarly, a survey of 320 patients with chronic arthritis treated with DMARDs in Pavia, Italy, found that none of the patients with a confirmed diagnosis of COVID-19 (n = 4) or a highly suggestive clinical picture (n = 4) or contact with a known COVID-19 patient (n = 5) developed severe respiratory complications or died [5].

A study in New York City by Haberman *et al.* described 86 COVID-19 patients with immune-mediated diseases and compared the characteristics of those who were hospitalized to those who were not (ambulatory patients) [6]. The mean age was slightly older in those who required hospitalization (50 vs 46 years in those who did not) and more males than females were hospitalized compared with ambulatory patients (50 vs 42%) [6]. Patients with rheumatoid arthritis (RA) had a higher frequency of hospitalization compared with Crohn’s, ankylosing spondylitis and psoriasis [6]. Therefore, it is not surprising that hospitalized patients were more likely to be receiving methotrexate (43%) versus ambulatory patients (15%) [6]. Those who received hydroxychloroquine (HCQ) had more hospitalizations (21%) versus ambulatory (7%), likely due to a diagnosis of RA [6]. This drug did not look protective but there was no comparison to the frequency of use of HCQ in RA patients who did not develop COVID-19 [6]. Glucocorticoids were more apt to be prescribed prior to hospitalization than in ambulatory patients (43 vs 15%), whereas TNF inhibitors were less likely to be received by patients requiring hospitalization (21 vs 49%) [6]. The numbers on other biologics and JAK inhibitors were too small to draw any conclusion [6].

A large multinational case series described the first 600 COVID-19 cases collected by the COVID-19 Global Rheumatology Alliance and identified risk factors for hospitalization for COVID-19 in patients with rheumatic diseases [7]. Similar to the findings by Haberman *et al.* [6], the median age was older in those that were hospitalized compared with nonhospitalized (62 vs 52 years) and more males than females were hospitalized compared with nonhospitalized patients (33 vs 26%) [7]. Most intriguingly, the report identified that age >65 years, comorbidities (namely diabetes, hypertension/cardiovascular, lung and renal diseases), >10 mg/day prednisone-equivalent glucocorticoids were associated with a higher risk of hospitalization for COVID-19 [7]. Conversely, TNF inhibitors were associated with a lower odd of hospitalization [7].

The studies to date have not had fully adequate control groups such as members of the community with rates of infection for age- and gender-matched people, and medications comparing those who developed COVID-19 to those who did not to see if certain diseases have a risk of more frequent infection and if the disease-modifying drugs are a risk, protective or irrelevant. Also, there could be biases in testing for COVID-19 more often in patients with immune-suppressive treatment.

## Medication shortages & their effect on patients

Many medications commonly used in the treatment of rheumatic diseases are currently being studied as potential treatments for COVID-19. HCQ and chloroquine were the first to receive substantial media attention. As a result, shortages of HCQ and/or chloroquine have been reported on Drug Shortages Canada [8] and American Society of Health-System Pharmacists [9] (as of 11 May 2020). Chloroquine was not available in Canada months before the pandemic, placing the supply of the medication in a precarious position.

Medication shortage is a complex health problem. Patients have anxiety that they will run out of medication, especially if it in some way prevents a severe outcome from COVID-19 infection. A scoping review found an increase in out-of-pocket drug costs, medication error rates, adverse events, mortality and patient complaints during times of drug shortage [10]. Thus, experts have expressed concerns over the limited availability of HCQ and chloroquine for patients with an approved indication such as lupus and RA [11,12]. Despite the efforts, on 28 March 2020, the US FDA issued an emergency use authorization for chloroquine and HCQ for the treatment of COVID-19. There are restrictions detailed in the authorization. Nonetheless, this hasty drug approval hindered patient adherence to immunosuppressive therapies. In the survey, 10% of the 197 patients who experienced some change in their care reported difficulty with obtaining their medication, especially HCQ [2]. Furthermore, a survey sent to 531 Canadian rheumatologists found that of the 134 rheumatologists who completed the survey, 81 (60%) had been contacted by patients or pharmacies regarding difficulty with HCQ access [13].

A disruption in maintenance therapy may lead to poorly controlled rheumatic disease, and high disease activity itself in rheumatic diseases, such as in RA, is associated with an increased risk of infection [14]. Governments have increased their supply of HCQ from several manufacturers in response to the shortage. The data of benefit for antimalarials in COVID-19 infection is sparse and large randomized trials are needed [15]. In fact, high-dose chloroquine had a higher death rate than low-dose chloroquine in a randomized controlled trial (RCT) [16].

Current evidence for the use of chloroquine and HCQ for treatment of COVID-19 remains limited and inconsistent. Therefore, efforts should be made to ensure that patients with previously existing medical indications be given priority access to the medication. The use of HCQ for COVID-19 should be restricted to clinical trials until further evidence is available.

## Immune modulators as COVID-19 treatments

Immunomodulating medications have been in the spotlight during this pandemic. This is due to the cytokine dysregulation and hyperinflammation seen in some severe COVID-19 cases [17].

Anakinra, an IL-1 inhibitor, has preliminary evidence for its safety and efficacy in patients with COVID-19. A retrospective cohort study compared the outcomes of 29 patients who received high-dose intravenous anakinra, noninvasive ventilation and standard treatment to those of the 16 patients who received only noninvasive ventilation and standard treatment [18]. Clinical improvements at 21 days were shown in 21 (72%) patients receiving high-dose anakinra and 8 (50%) patients receiving standard treatment [18]. At 21 days, three (10%) patients receiving high-dose anakinra died versus seven (44%) patients receiving standard treatment [18]. The immune modulator is currently being investigated in at least 11 COVID-19 related clinical trials registered on ClinicalTrials.gov.

Multiple IL-6 inhibitors, such as tocilizumab, sarilumab and siltuximab, are also being investigated as COVID-19 treatments. Tocilizumab has the most preliminary evidence so far. A retrospective study with 21 patients in China showed that tocilizumab is effective in reducing mortality in severe and critical COVID-19 patients [19]. Another retrospective study in China with 15 patients with COVID-19 treated with tocilizumab suggested that it is a possible treatment option for patients at risk of cytokine storms [20]. Many more tocilizumab clinical studies are currently underway worldwide. Data involving siltuximab and sarilumab in COVID-19 are pending peer review or still under investigation.

Baricitinib, a JAK inhibitor, is predicted to block SARS-CoV-2 receptor-mediated endocytosis into pulmonary epithelial cells, as well as excessive JAK-dependent cytokine signaling [21]. An open-label study with 12 patients with oral baricitinib added to ritonavir/lopinavir therapy showed promising results as all patients improved at 2 weeks and required no ICU admission [22]. At least, five clinical trials involving baricitinib have been registered on ClinicalTrials.gov.

TNF inhibitors, such as adalimumab, have also garnered some attention [23]. There is one study evaluating adalimumab in COVID-19 registered in the Chinese Clinical Trial Registry 2000030089. Several other biologic and antirheumatic drugs have been identified to be theoretically helpful in the treatment of COVID-19 including colchicine, IL-17 inhibitors, IL-12 and IL-12/23 inhibitors, mycophenolate mofetil, azathioprine, methotrexate, cyclosporine, tacrolimus and other treatments [24] (accessed on 18 May 2020). There is speculation that anticytokine therapies used in rheumatology and dermatology such as IL-17, IL-12/23 and IL-23 inhibitors are safe to use during COVID-19 and may have some potential benefit theoretically [25,26].

For all these medications, there are currently insufficient data to recommend either for or against their use for the treatment of COVID-19. Supplementary Table 1 summarizes potential immune modulator treatments for COVID-19.

## Conclusion

The COVID-19 pandemic is a rapidly evolving situation, and information on the topic is released and revised as the situation unfolds each day. Current evidence shows that rheumatology patients do not seem to have worse outcomes despite immune suppression. Many immunomodulating medications are being studied as treatments of COVID-19, which can lead to negative impacts on the health and wellbeing of patients treated with immunotherapies secondary to medication shortages. It is important to still follow steps that have been put in place for years to safeguard proper medication development and usage. These steps require valid randomized controlled trials for approving medications and prescribing treatments. As many have expressed previously, immunosuppressive medications are not without adverse effects and, thus, these medications should be used only with careful considerations and be allocated to patients who have proven to benefit from these therapies [12]. There are likely to be long-term consequences for patients with immune-mediated diseases after COVID-19 including chronic stress/anxiety, depression and pain, an increase in fibromyalgia and post-traumatic stress disorder and flaring of immune-mediated diseases in some patients due to financial and health stressors.
