# Supplementary material for: How COVID-19 affects patients receiving anticytokine and JAK inhibitors in rheumatology and dermatology
Source: Immunotherapy. 2020 Jul 9:10.2217/imt-2020-0153. doi: 10.2217/imt-2020-0153 (PMC7346716; doi:10.2217/imt-2020-0153)
Supplement: Supplementary file 1 [file imt-2020-0153_supp_table_1.doc]

| **Drug Name** | **Proposed Mechanism of Action** | **Current Clinical Data for COVID-19** |
| --- | --- | --- |
| *Interleukin (IL)-1 inhibitor* | | |
| anakinra | Blocks IL-1 binding to receptor | - Retrospective cohort study with 29 patients who received high-dose intravenous anakinra, non-invasive ventilation, and standard treatment vs 16 patients who received only non-invasive ventilation and standard treatment show preliminary data supporting the safety and efficacy of anakinra [18]. |
| *Interleukin (IL)-6 inhibitor* | | |
| tocilizumab | Blocks IL-6 binding to receptor | - Retrospective study with 21 patients in China showed that tocilizumab is effective in reducing mortality in severe and critical COVID-19 patients [19]  - Retrospective study with 15 patients in China showed that tocilizumab is a possible treatment option for COVID-19 patients at risk of cytokine storms [20] |
| sarilumab |  | No published data |
| siltuximab |  | No published data |
| *Janus kinase inhibitor* | | |
| baricitinib | Reduce SARS-CoV-2 receptor-mediated endocytosis, as well as reduce excessive cytokine signaling [21] | - Open-label study with 12 patients given oral baricitinib in addition to ritonavir/lopinavir therapy showed that all patients improved at 2 weeks and none required ICU admission [22] |
| *Anti-tumour necrosis factor (TNF) antibodies* | | |
| adalimumab | Reduce SARS-CoV-2 penetration into host cell and reduce lung injury caused by mechanism related to TNFα production [23] | No published data |

Supplemental Table 1. Summary of preliminary data on anti-cytokine and Janus kinase inhibitor medications used in rheumatology for the potential treatment in COVID-19.
